# Single-row vs. double-row refixation of the subscapularis tendon after primary anatomic shoulder arthroplasty

**DOI:** 10.1007/s00402-020-03423-5

**Published:** 2020-04-28

**Authors:** Yacine Ameziane, Kristian Nikolaus Schneider, Georg Gosheger, Annika Mischke, Dominik Schorn, Carolin Rickert, Dennis Liem

**Affiliations:** 1 Department of Orthopedics and Tumor Orthopedics, University Hospital Muenster, Albert-Schweitzer-Campus 1a, 48149 Muenster, Germany; 2 Orthopaedic Practice Clinic, Muenster, Germany; 3 Sporthopaedicum Berlin, Berlin, Germany

**Keywords:** Shoulder arthroplasty, Subscapularis, Shoulder, Ultrasound, Tenotomy, Refixation

## Abstract

**Background:**

The postoperative integrity of the subscapularis tendon after primary anatomical shoulder arthroplasty has a significant effect on postoperative results. A transosseus Single Row Refixation technique (SRR) has shown up to 30% of partial tears in literature, a modified Double Row Refixation technique (DRR) has biomechanically shown a significantly reduced tear rate, but is yet to be proven in a clinical setting. Thus, we compared the SRR to the DRR technique using clinical outcome parameters and ultrasound examination.

**Materials and methods:**

36 patients (40 shoulders; 20f, 16 m; øage: 66 years) were included in our retrospective cohort study. 20 shoulders were treated with the SRR technique (12f, 8 m; FU ø40.9 months) and 20 with the DRR technique (11f, 9 m; FU ø31.6 months). The SRR was performed with three to five transosseus mattress sutures. DRR consisted of two medial placed transosseus sutures and four laterally placed single tendon-to-tendon sutures. The postoperative subscapularis integrity was evaluated by ultrasound examination, the clinical outcome was assessed with the Constant–Murley Score (CS) and the American Shoulder and Elbow Surgeons Score (ASES).

**Results:**

The subscapularis tendon was intact in 14 patients (70%) after SRR, whereas 18 patients (90%) treated with the DRR demonstrated a sonographically intact postoperative subscapularis tendon. The CS was 61.4 points in the SRR cohort and 67.3 points in the DRR cohort (*p* = 0.314). No significant differences were found in both cohorts preoperative (øSRR: 21.3 points; øDRR: 16.2 points, *p* = 0.720) and postoperative absolute ASES Scores (øSRR: 70.2 points; øDRR: 73.0 points, *p* = 0.792). However, the DRR cohort showed a statistical tendency to a higher postoperative ASES increase than the SRR cohort (øSRR-ASES increase: 48.9 points; øDRR-ASES increase: 56.8 points, *p* = 0.067).

**Conclusion:**

The results of this study show that application of the DRR technique can significantly reduce the total rate of postoperative subscapularis tears what effects a clinical tendency towards higher ASES improvements and a better range of motion compared to the SRR technique.

## Introduction

Shoulder arthroplasty has become a widely used and acknowledged procedure with good to excellent mid- to long-term results for patients with primary or secondary glenohumeral osteoarthritis [20, 22]. The deltopectoral approach is commonly considered as the standard approach for shoulder arthroplasty including subscapularis take-down and refixation. The subscapularis is the most powerful muscle of the rotator cuff acting as an internal rotator, a depressor and a stabilizer of the glenohumeral joint and the refixation remains a key area of concern as insufficient refixation can negatively impact the postoperative outcome leading to anterior instability [4, 15, 18]. Several studies have already tested different surgical techniques of subscapularis refixation and reported postoperative signs of clinical subscapularis insufficiency in 9–67% and at least partially compromised structural integrity in 13–47% of cases [2, 5, 6, 8, 12–14, 16, 17].

Thus, the best surgical technique of subscapularis refixation still remains unclear: although the transosseus Single Row Refixation (SRR) constitutes a long established technique, recent literature has shown postoperative partial tears of the subscapularis tendon in up to 30% of all cases [13]. In an effort to improve structural integrity of the SRR, the Double Row Refixation (DRR) was biomechanically introduced by Ahmad et al. [1]. Their cadaveric study presents a significantly higher fixation strength of the DRR technique under cyclic loading compared to the simple transosseus SRR technique. However, literature lacks valuable clinical studies that transmit these biomechanical findings into a clinical setting. In our clinical setting, we changed our technique to the DRR technique in an effort to improve structural and possibly clinical results. We conducted this study to compare our earlier results with the SRR technique to the DRR technique regarding the postoperative integrity of the subscapularis tendon and the subsequent clinical outcome. The hypothesis of this study was that the DRR technique would result in a higher rate of intact subscapularis tendons after anatomic shoulder arthroplasty and provide at least equally good clinical results.

## Materials and methods

Our study is based on two datasets—one ‘historical’ SRR cohort and a ‘current’ DRR cohort—comprising 20 shoulders each. All patients received primary hemiarthroplasty (*n* = 16) or total shoulder arthroplasty (TSA) (*n* = 24) in our clinic. The SRR cohort was surgically treated between 05.11.2001 and 09.02.2005 while DRR cohorts patients were operated between 02.10.2009 and 17.12.2014. The indication for surgery was either primary osteoarthritis (*n* = 34) or necrosis of the humeral head (*n* = 6). All patients were surgically treated by the same orthopedic consultant. Patients, who displayed posttraumatic arthritis, rheumatic arthritis or cuff tear arthropathy were excluded from this study as well as patients with a history of previous shoulder surgery.

**Fig. 1:**
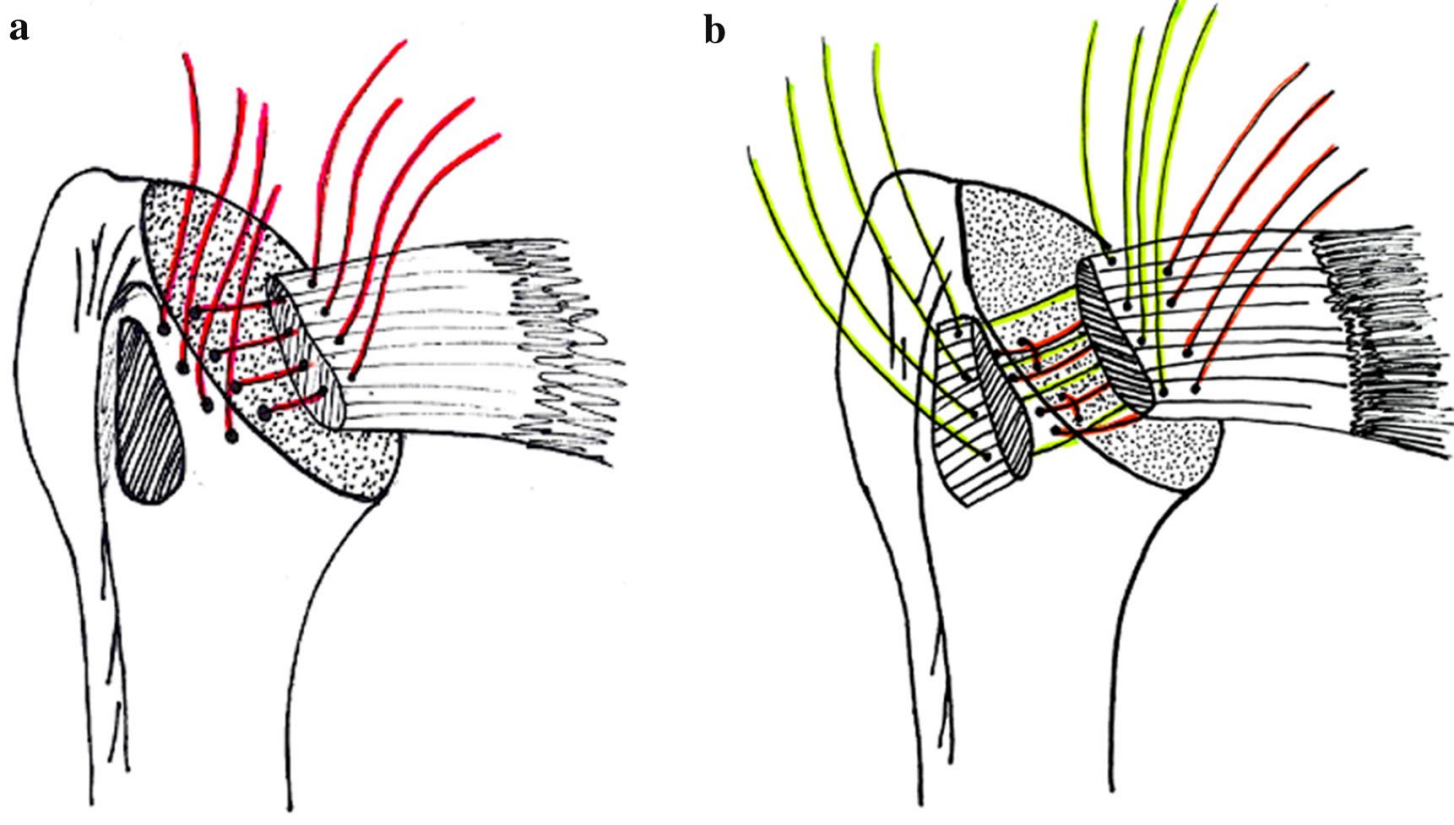
**a** Single Row Refixation. **b** Double Row Refixation

The ‘historical’ SRR cohort consisted of 19 patients (20 shoulders; 10f, 9m), with an average age of 69.5 years (± 8.41) and a follow-up of 41.0 months (± 12.31). 12 patients were operated on their dominant shoulder and eight on their non-dominant one. One patient received bilateral shoulder arthroplasty. In this group, subscapularis refixation was performed using the SRR technique: Three to five transosseus sutures (No. 2 Ethibond®, Ethicon, Inc., Somerville, NJ, USA) were placed through the proximal humerus before implanting the prosthesis and re-attached to the tendon via mattress stitching technique (Fig. 1a; Table 1).

**Table 1:** Demographics

	SRR ( ±)		DRR ( ±)	*p*
Age (years)	69.5 (8.41)		63 (10.26)	0.072
FU (months)	41 (12.31)		31.6 (15.5)	0.014
F/M	10 / 9		9 / 8	
Dominant/non-dominant shoulder treated	12 / 8		13 / 7	
TSA	12		12	
HAS	8		8	

*FU* follow-up, *f / m* female/ male, *TSA* total shoulder arthroplasty, *HAS* hemi shoulder arthroplasty

The ‘current’ DRR cohort consists of 17 patients (20 shoulders; 9f, 8m). Patients had an average age of 63 years (± 10.26). The average follow-up was 31.6 months (± 15.5). The dominant shoulder was affected in 13 cases, the non-dominant one in seven cases. Three patients received bilateral shoulder arthroplasty. In this cohort, the subscapularis tendon was re-attached using a DRR technique with a medial and a lateral row: The medial row consisted of two transosseus sutures (No. 2 Ethibond®, Ethicon, Inc., Somerville, NJ, USA) and the additional lateral row consisted of two to four tendon-to-tendon sutures (Fig. 1b; Table 1).

### Operative technique

All patients were operated in beach-chair position under general anesthesia. The deltopectoral approach was used to access the glenohumeral joint. The coracohumeral ligament was released while the coracoacromial ligament remained intact. After exposition of the subscapularis, two tagging sutures were placed through the medial part of the tendon, while other visible anatomic landmarks, such as the rotator interval, bicipital groove and circumflex vessels, were identified. The circumflex vessels were ligated and a tenotomy of the subscapularis tendon and capsule was performed medial to the lesser tuberosity. The tenotomy started next to the rotator cuff interval and ended next to the onset of M. latissimus dorsi, which was incised. In the SRR cohort the tenotomy was performed as a peel-off next to the insertion of the subscapularis with no tendon stump remaining on the lesser tuberosity. In the DRR cohort, the tenotomy was medialized about 5 mm to create a tendon stump for the placement of the lateral tendon-to-tendon sutures. After the rotator cuff interval was opened-up to the base of the coracoid, the glenohumeral ligaments were released from superior to inferior. The hypertrophic synovia and capsule were intraarticularly resected as needed to allow sufficient access to the glenoid.

While one type of prosthesis (Univers I®, Arthrex, Naples, FL, USA) was used in the SRR cohort, two different types of prosthesis (Global ap®, Depuy, Raynham, Massachussets, USA; Eclipse®, Arthrex, Naples, Florida, USA) were implanted in the DRR cohort. 24 shoulders were treated with TSA, using a cementless glenoid interference screw in 12 cases and a cemented glenoid component in another 12 cases (pegged: 9; keeled: 3). 16 shoulders received hemiarthroplasty. After implanting the prosthesis, the subscapularis tendon was re-attached to the lesser tuberosity with the SRR and DRR technique, respectively*.*

Postoperative protocols were identical in both groups. The operated shoulder was immobilized in a sling for four weeks. Passive range of motion (ROM) was limited to 90° flexion, 90° abduction and 20° of external rotation for the first six weeks after surgery. Subsequently, passive and active ROM were encouraged whereas heavy physical activity and sports were allowed after six months.

### Clinical assessment

All patients underwent physical examination by one of two independent orthopedic surgeons. The obtained clinical results were evaluated through pre- and postoperative use of the American Shoulder and Elbow Surgeon Score (ASES). The Constant–Murley Score (CS) was postoperatively calculated for both shoulders.

All patients received postoperative radiographic examination with a true anterior posterior (true-a.p.) and Y view. The true-a.p. view was used to evaluate the acromio-humeral distance (AHD). An AHD of less than 5 mm was taken as a sign for rotator cuff tear arthropathy [10]. Axillary views were available in 15 cases of the SRR cohort and in 19 cases of the DRR cohort. According to a classification described by Habermeyer et al. all axillary views were analyzed for anterior dislocations as a potential sign for subscapularis insufficiency [9]. An anterior deviation of more than 5 mm to the glenoid center line was defined as an anterior decentralization of the humeral head [9].

**Fig. 2:**
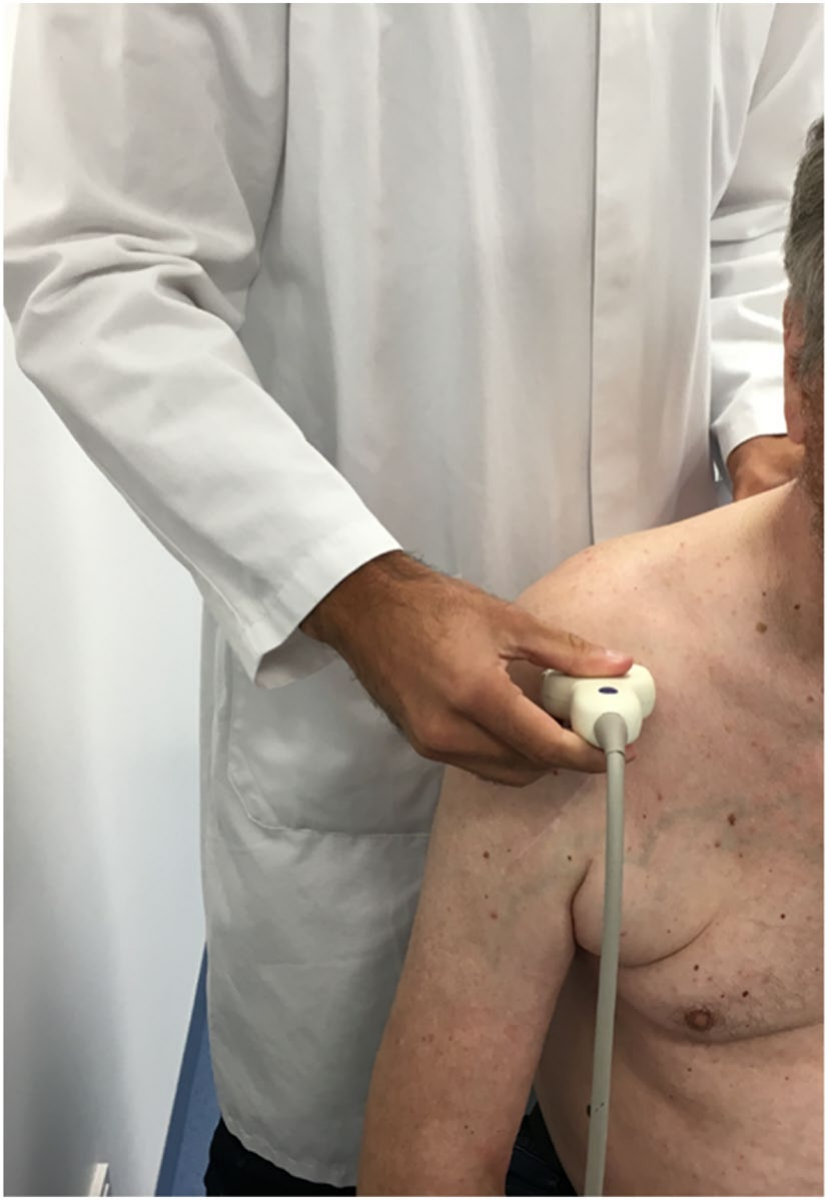
Ultrasound examination

All patients underwent an ultrasound examination of the operated and contralateral shoulder to assess the postoperative integrity of the subscapularis tendon (Fig. 2). Indicators of a fully torn tendon were absence and/or lack of mobility of the tendon during humeral rotation [11]. A partial tear was considered when less than 50% of the original subscapularis tendon thickness was present.

### Statistical analysis

Statistical analysis was performed using SPSS 24.0 (SPSS Science, Chicago, IL, USA). The data were checked for normative variance with the Kolmogorow–Smirnow Test; and if normative variance was found, the *t*-test or the Mann–Whitney *U* test were used for comparison and analysis. The level of significance was set at *p* = 0.05.

## Results

### Subscapularis integrity

Of the 20 shoulders in our SRR cohort, six shoulders (30%) displayed sonographic signs of a partial subscapularis tear. 14 shoulders (70%) showed adequate tendon thickness during ultrasound examination and passive movement of the tendon/muscle during physical examination.

Of the 20 shoulders in our DRR cohort, 18 shoulders (90%) demonstrated an intact subscapularis tendon with complete integrity. One shoulder (5%) was diagnosed with a partial tear. In one case (5%) the subscapularis tendon was completely torn. No traumatic events that could have affected the subscapularis were reported in any of the aforementioned cases.

### Objective subscapularis function

There was no significant difference (*p* = 0.314) between the postoperative CS of the SRR cohort (61.4 points) and the DRR cohort (67.3 points; Table 2).

**Table 2:** Objective clinical results

	SRR ( ±)	DRR ( ±)	*p*
Pain	13.2 (2.1)	11.5 (3.1)	0.076
ADL	12.4 (4.5)	14.6 (3.2)	0.157
ROM	27.3 (7.4)	29.8 (7.5)	0.445
Strength	8.5 (4.1)	11.4 (6.5)	0.445
Constant–Murley score (points)	61.4 (15.1)	67.3 (12.5)	0.314
Constant–Murley score (%)	72.4 (17.3)	78.5 (14.3)	0.461

*ADL* activities of daily living, *ROM* range of motion

However, comparison of the pre- to postoperative improvement in the ASES Scores of both cohorts showed a tendency towards statistical significance: The SRR cohort improved by an average of 39.3 points, whereas the DRR cohort increased by an average of 54.9 points (*p* = 0.067; Table 3). These differences are further underlined by comparison of absolute ASES Scores: Pre-surgery, the average ASES Score of the SRR cohort (21.3 points) was better compared to the DRR cohort (16.2 points, *p* = 0.720). Postoperatively, however, the average ASES Score of the DRR cohort (73.0 points) exceeded that of the SRR cohort (70.2 points, *p* = 0.792).

**Table 3:** ASES score

	SRR ( ±)	DRR ( ±)	*p*
Preoperative	21.3 (17.8)	16.2 (11.7)	0.720
Postoperative	70.2 (19.8)	73.0 (14.1)	0.792
ASES increase	39.3 (22.7)	54.9 (18.0)	0.067

### Range of motion

Postoperatively, DRR patients displayed a significantly higher (*p* = 0.016) range of motion in abduction (ø148°) than SRR patients (ø113°). Correspondingly, DRR patient’s flexion and external rotation exceeded that of SRR patients (ø159° versus ø138°, *p* = 0.176 and ø48° versus ø43°, *p* = 0.762). A tendency (*p* = 0.167) towards improved internal rotation was evident in the DRR cohort: While on average SRR patients were able to move their hand up to S1, the DRR cohort could, on average, reach up to Th12.

### Radiologic examination

No significant differences could be found in the AHD. The average preoperative AHD was 9.4 mm (range 7.0–12.0 mm) in the SRR cohort and 10.6 mm (range 7.0–15.8 mm) in the DRR cohort. Postoperatively, no signs for a rotator cuff tear arthropathy were present in both cohorts: The SRR group displayed an average AHD of 9.30 mm (range 5.0–14 mm, *p* = 1) the DRR cohort of 10.2 mm (range 5.6–16.7 mm, *p* = 0.191). Regarding decentralization, seven patients (35%) in the SRR cohort were anteriorly decentralized more than 5 mm. Four of these seven patients (57%) were diagnosed with a partial tear in ultrasound examination. In the DRR cohort, the axillary X-rays of four patients (20%) displayed signs of anterior decentralization. While three of these patients had an intact tendon in ultrasound examination, one patient (20%) had shown sonographic signs of a partial subscapularis tear.

## Discussion

Due to its stabilizing and rotative effect the refixation of the M. subscapularis tendon crucially affects the postoperative outcome in shoulder arthroplasty [2]. The purpose of this study is the comparison of two different subscapularis refixation techniques: The SRR and the DRR technique. Subjective and objective parameters were obtained to assess the postoperative subscapularis tendon integrity and its impact on the clinical outcome.

The loss of function of the subscapularis is a well-known problem after shoulder arthroplasty. Miller et al. compared a large SRR cohort (32 patients) to nine patients who received a simple soft tissue tendon-to-tendon repair after subscapularis tenotomy [14]. Although the clinical results of the SRR treated patients exceeded those of soft tissue tendon-to-tendon refixation, the SRR cohort still demonstrated positive clinical signs of subscapularis insufficiency in over 66% of all cases. They concluded that the subscapularis take-down is “a seldom recognized problem”. In contrast, Caplan et al. published good to excellent clinical results of a soft tissue tendon-to-tendon refixation with negative postoperative signs for subscapularis insufficiency in 91–100% in 43 patients [6]. However, these results do not correspond with the findings of Miller et al. nor with similar studies like Jackson et al. who reported positive signs of a subscapularis tear in more than 40% of all patients treated with a soft tissue tendon-to-tendon refixation [12].

Armstrong et al. reviewed 30 shoulders regarding the subscapularis integrity after shoulder arthroplasty and a SRR with a mean age of 70.4 years and follow-up of 20.4 months [2]. Using ultrasound, an intact tendon was present in 87% of all cases and a full torn tendon in four patients (13%). Our SRR cohort that is comparable aged (Armstrong et al.: ø70.4 years; et al.: ø69.5 years) and presents a longer follow-up (Armstrong et al.: ø20.4 months; et al.: ø41.0 months) showed improvable results with partial ruptures found in six cases (30%). In contrast only two patients (10%) of our DRR cohort (age: ø63 years; FU: ø31.6 months) demonstrated a partial or complete tear, postoperatively.

As a consequence of poor clinical results, the predominant SRR technique was challenged by many surgeons and evolved to a DRR technique that was first biomechanically tested by Ahmad et al. in a cadaveric study proving that the SRR technique has deficits regarding the strength of fixation. In addition, the DRR technique achieves a higher grade of “tendon-to-humerus contact” after cyclic loading which let the authors presume a greater final strength after biological healing [1]. De Franco et al. established three criteria for ideal tendon healing: high initial fixation strength, minimal gap formation at the interface and stability until healing is completed [7]. All of those are achieved by the DRR technique.

Armstrong et al. published retrospective results of a DRR technique for subscapularis refixation after TSA in 30 shoulders [3]. The average ASES Score improved significantly from pre- to postoperatively from 45.3 to 76.8 points. Two complete ruptures (7%) were found in 30 subscapularis tendons during ultrasound examination—similar results compared to our postoperative DRR tear rate of 10% (one complete rupture, one partial tear; preoperative øASES: 16.2 points to postoperative øASES: 73.0 points).

Evaluation of radiographic examination was also performed and correlated with structural integrity of the subscapularis. We could show moderate to low correlation between anterior decentralization and subscapularis integrity: While 57% of SRR patients simultaneously displayed anterior decentralization and a partial subscapularis tear, only 20% of DRR patients showed such results. The degree of correlation between anterior decentralization and subscapularis integrity is further limited by the fact that, on a regular basis, decentralization of the humeral head is found preoperatively and cannot always be corrected postoperatively.

To our knowledge, this study is the first to confirm Ahmad et al.’s biomechanical findings in a clinical setting and to directly compare the tendon integrity following DRR-based surgeries versus SRR techniques. A further strength of this study is the high comparability of the two cohorts of patients—the only notable difference being the suture technique used for refixation. Finally, this study has some limitations. Firstly, the small number of patients and the inclusion of patients with osteoarthritis and humeral head necrosis as primary indications. Even though we would have wished the number of patients to be larger, the number of patients is comparable to other publications in this field. Literature is lacking studies that show postoperative differences between osteoarthritis and humeral head necrosis as indications for primary shoulder arthroplasty [21]. This study includes patients treated with hemi- or total shoulder arthroplasty. However, to our knowledge, no studies have yet been published indicating different subscapularis healing rates between hemi- or total shoulder arthroplasty. Findings across the two methods are thus considered comparable. Secondly, ultrasound–on which this study relied–provides only little information on muscle degeneration, which is very important for the subscapularis function. Ultrasound examination is nevertheless evaluated and described as a useful method of imaging the rotator cuff after shoulder arthroplasty [19]. Further, no specific tests for subscapularis strength and function were performed during clinical examination. This is in line with a previous study that presented low sensitivity, specificity and positive predictive value for clinical tests to assess the subscapularis integrity and function [2]. Thirdly, the ASES Score was the only surveyed parameter that allowed a pre- and postoperative comparison of the two cohorts. Thus, this study constitutes a platform for further research, e.g. for the use of more comparative methods as well as radiographic measures other than ultrasound to assess effects on muscle degeneration following different subscapularis refixation techniques.

## Conclusion

This study clinically confirms that a modification of the intraoperative subscapularis refixation technique after anatomic shoulder arthroplasty from a SRR to a DRR technique can considerably reduce the total rate of postoperative subscapularis pathologies. We could sonographically confirm a reduction of incomplete healing from 30 to 10%. Clinically we found a tendency towards higher ASES Score improvements and better range of motion in the DRR group.
